# Online Search Trends Influencing Anticoagulation in Patients With COVID-19: Observational Study

**DOI:** 10.2196/21817

**Published:** 2021-08-31

**Authors:** Amy P Worrall, Claire Kelly, Aine O'Neill, Murray O'Doherty, Eoin Kelleher, Anne Marie Cushen, Cora McNally, Samuel McConkey, Siobhan Glavey, Michelle Lavin, Eoghan de Barra

**Affiliations:** 1 Department of Infectious Diseases Beaumont Hospital Dublin Ireland; 2 Department of Haematology Beaumont Hospital Dublin Ireland; 3 Department of Anaesthesiology Beaumont Hospital Dublin Ireland; 4 Department of Pharmacy Beaumont Hospital Dublin Ireland; 5 Department of International Health and Tropical Medicine Royal College of Surgeons Ireland Dublin Ireland; 6 Irish Centre for Vascular Biology School of Pharmacy & Biomedical Sciences Royal College of Surgeons Ireland Dublin Ireland

**Keywords:** COVID-19, coronavirus, online search engines, anticoagulation, thrombosis, online influence, health information dissemination

## Abstract

**Background:**

Early evidence of COVID-19–associated coagulopathy disseminated rapidly online during the first months of 2020, followed by clinical debate about how best to manage thrombotic risks in these patients. The rapid online spread of case reports was followed by online interim guidelines, discussions, and worldwide online searches for further information. The impact of global online search trends and online discussion on local approaches to coagulopathy in patients with COVID-19 has not been studied.

**Objective:**

The goal of this study was to investigate the relationship between online search trends using Google Trends and the rate of appropriate venous thromboembolism (VTE) prophylaxis and anticoagulation therapy in a cohort of patients with COVID-19 admitted to a tertiary hospital in Ireland.

**Methods:**

A retrospective audit of anticoagulation therapy and VTE prophylaxis among patients with COVID-19 who were admitted to a tertiary hospital was conducted between February 29 and May 31, 2020. Worldwide Google search trends of the term “COVID-19” and anticoagulation synonyms during this time period were determined and correlated against one another using a Spearman correlation. A *P* value of <.05 was considered significant, and analysis was completed using Prism, version 8 (GraphPad).

**Results:**

A statistically significant Spearman correlation (*P*<.001, *r*=0.71) was found between the two data sets, showing an increase in VTE prophylaxis in patients with COVID-19 with increasing online searches worldwide. This represents a proxy for online searches and discussion, dissemination of information, and Google search trends relating to COVID-19 and clotting risk, in particular, which correlated with an increasing trend of providing thromboprophylaxis and anticoagulation therapy to patients with COVID-19 in our tertiary center.

**Conclusions:**

We described a correlation of local change in clinical practice with worldwide online dialogue and digital search trends that influenced individual clinicians, prior to the publication of formal guidelines or a local quality-improvement intervention.

## Introduction

Since late 2019, the knowledge of the clinical sequelae of COVID-19 has increased largely through the rapid dissemination of information through various information platforms. In addition to asymptomatic infection, SARS-CoV-2 can cause a broad range of symptoms, from mild coryzal symptoms to neurological and gastrointestinal presentations and, most worryingly, severe acute respiratory failure. In the early months of 2020, reports of a high incidence of COVID-19–related coagulopathy circulated online, with anecdotal evidence and case reports emerging initially. This was soon followed by marked increases in clinical thrombosis, including deep vein thromboses, pulmonary emboli [1,2], and microthrombi in pulmonary vasculature in postmortem pathological studies [3]. Clinical debate ensued surrounding the best strategy to prevent and treat COVID-19–associated coagulopathy. By the middle of April 2020, an international position paper recommended the provision of adequate thromboprophylaxis in hospitalized patients with COVID-19 [4]. Debate began on the use of therapeutic anticoagulation in the absence of confirmed thrombosis, or an intermediary dose of low-molecular-weight heparin as a thromboprophylaxis in this cohort. The risk-benefit ratio of bleeding versus clotting had to be taken into consideration, especially as some patients displayed signs of disseminated intravascular coagulopathy. However, the role of online search trends and online health information tools in the spread of this information has yet to be assessed.

The COVID-19 pandemic has rapidly evolved, spreading throughout the globe from its origin in China. The growing need to quickly report clinical findings, guidance, and recommendations became paramount as the seriousness of the situation unfolded. Chinese reports and studies were soon followed by those of our Italian colleagues. Italy became the first country to provide a European angle on treatment and management of COVID-19. Dramatic changes occurred worldwide, forcing the public into the safety of their homes. Social distancing measures, including quarantine, business closures, and travel bans, meant the ability for academic, clinical, and public discourse was limited. In order to gain information on COVID-19, the most recent updates, and suggested management, clinicians began engaging with online and social media platforms, ones that have already been described in the literature [5].

Infodemiology studies have already shown that search trends are useful parameters in the measurement of this pandemic; for example, search trends for symptoms correlating with disease outbreak [6]. Similarly, infodemiology metrics for search trends on Google across European countries showed a strong correlation between COVID-19 cases locally and worldwide, identifying new avenues for online disease surveillance and response efforts [7].

Here, we investigate the power of search trends as representative of discussion and dissemination of clinical information and recommendations online as well as how trending themes and discussion have influenced clinical practice in our local center.

## Methods

**Figure 1:**
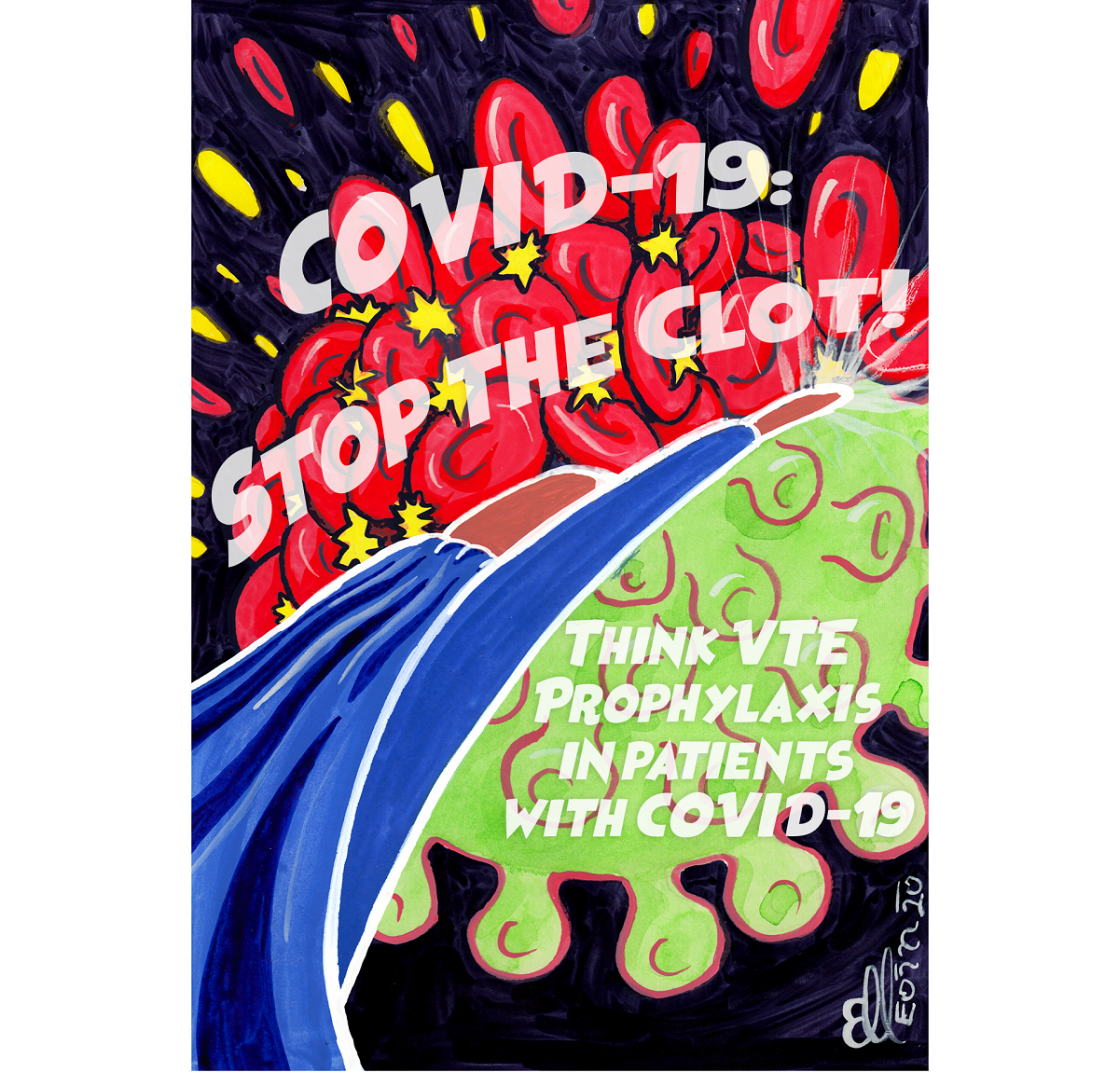
Local infographic used to encourage venous thromboembolism (VTE) prophylaxis in patients with COVID-19, designed by Dr Eoin Kelleher (@eoinkr) and circulated in print and on digital platforms throughout our tertiary center.

We conducted a retrospective audit of patients who tested positive for COVID-19 by real-time reverse transcription polymerase chain reaction at Beaumont Hospital in Dublin, Ireland, a tertiary 820-bed hospital, from March 1 to May 31, 2020. The audit received approval from the audit department and the research ethics committee of Beaumont Hospital. All patients were followed until the June 20, 2020. Data regarding venous thromboembolism (VTE) risk was assessed using the Padua Prediction Score system. The appropriate prescribing of thromboprophylaxis or anticoagulation therapy within 24 hours of admission or 24 hours of a positive COVID-19 result were collected. Three local interventions in the hospital were noted as key events that influenced anticoagulation and thromboprophylaxis guidelines for patients with COVID-19. These three interventions included the following: a COVID-19 teaching session by the coagulation specialist, a consultant hematologist (attending); a hospital-wide email with VTE prophylaxis guidelines; and hospital-wide circulation of an infographic about VTE prophylaxis in patients with COVID-19 (Figure 1).

Data from Google Trends were retrieved online in comma-separated values (CSV) format and used to compile a representative trend of worldwide searches for “COVID-19” and synonyms (eg, “coronavirus” and “covid”), together with the following terms: “anticoagulation,” “VTE prophylaxis,” “thrombosis,” “clots,” and “clotting.” The search results were then summated. Data collection included results from February 29 until May 31, 2020. The data were worldwide data, rather than European or Irish Google Trends data, as Google search results at the time were very scarce in individual countries. The Google Trends data were being used to infer online activity on numerous social media platforms during the early period of the COVID-19 pandemic. Data were analyzed using Prism, version 8 (GraphPad), according to nonparametric Spearman correlations. A *P* value of <.05 was considered statistically significant.

## Results

A total of 399 patients consecutively diagnosed with COVID-19 were reviewed during the study period. The median age of the patients was 70 years (IQR 27, range 21-99), the majority were male (247/399, 61.9%) and Caucasian (360/399, 90.2%), and 81.5% (325/399) had underlying comorbidities. On admission, patients were assessed for thrombotic and bleeding risks. The median Padua Prediction Score was 4 (IQR 4, range 1-12). A total of 14.3% of patients (57/399) were on anticoagulation therapy for thrombotic or cardiac disorders on admission. A total of 55.1% of patients (220/399) had commenced standard thromboprophylaxis doses of heparin within the first 24 hours of admission or within 24 hours of a positive COVID-19 result. A total of 89.1% of anticoagulation prescriptions (196/220) were correctly adjusted for BMI or renal function within the same admission time frame.

**Figure 2:**
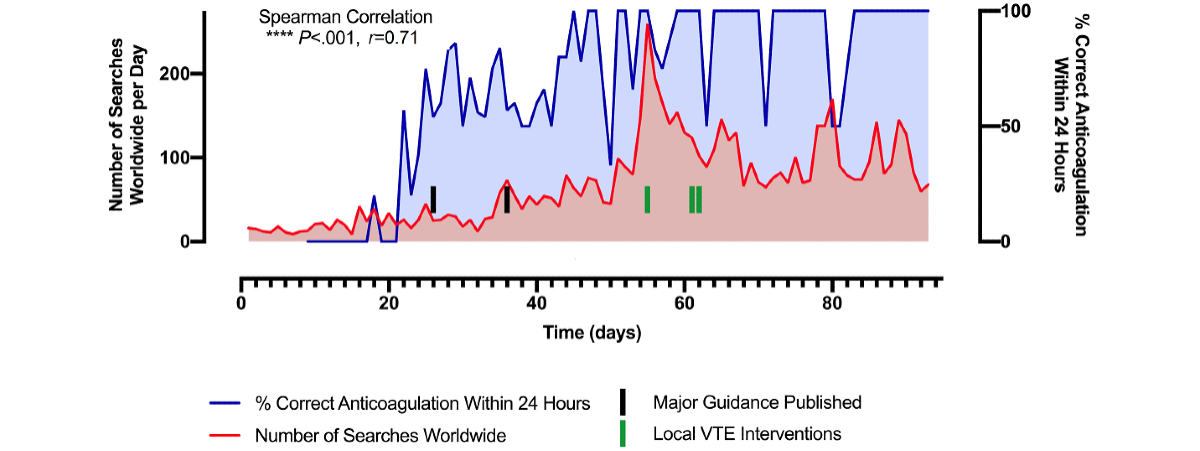
Run chart of daily online searches relating to COVID-19 and anticoagulation (blue, left axis) and the percentage of correct anticoagulation medications administered to the cohort of patients with COVID-19 (red, right axis). Black dashes indicate the publication of two major international, interim, guidance papers for patients with COVID-19 [4,8], and green dashes indicate three local venous thromboembolism (VTE) interventions in our tertiary center.

A total of 6361 individual searches worldwide were collated from Google Trends over the study period relating to the COVID-19 pandemic and thrombosis. The percentage of patients with COVID-19 who were on appropriate anticoagulation therapy was graphed as a percentage of the total number of patients during the admission period (Figure 2 [4,8]). A statistically significant Spearman correlation (*P*<.001, *r*=0.71) was found between the two data sets. Our study demonstrated that online searches and discussion, dissemination of information, and Google search trends relating to COVID-19 and clotting risk, in particular, correlated with an increasing trend of providing thromboprophylaxis and anticoagulation therapy to patients with COVID-19 in our tertiary center. Following the publication of two major guideline papers [4,8] (Figure 2) and three local VTE interventions, there was significant improvement in VTE prophylaxis among our cohort of patients with COVID-19 infection (Figure 2).

## Discussion

### Principal Findings

Mavragani and colleagues demonstrated that Google Trends search queries correlated strongly with COVID-19 cases and COVID-19 deaths, but also that their correlations were most accurate during the initial months of a region’s outbreak [7]. Our correlation similarly reflected search trends that correlated with a change in clinical practice locally. However, in 2015, Narayanaswami et al published a longitudinal study of the effects of traditional versus novel information dissemination (online resources, social media platforms, etc) on implementing and disseminating clinical guidelines [9]. They found no additional benefit from online resources in increasing awareness and implementation of clinical guidelines, though we hypothesize that this question may need revisiting if considering the developments in online resources since 2015 and the unique global impact and increased reliance on online assets during the COVID-19 pandemic.

Online learning platforms, information and news sources, as well as social media do impact and influence a subgroup of physicians that use those resources [5], and it is not outlandish to suggest that the number of clinicians engaging in online health information discussions has increased during the COVID-19 pandemic. The consequences of this can be positive; for example, the use of our local VTE prophylaxis increased significantly within our cohort prior to the three local VTE anticoagulation interventions, most likely secondary to two major interim guidance documents being published and significant international online discussion surrounding COVID-19 coagulopathy. However, as seen with the rapid and somewhat premature use of hydroxychloroquine in patients with COVID-19 or the hasty alarm surrounding ibuprofen use in COVID-19 cohorts, in an evolving pandemic, knee-jerk medical management and interventions can occur without stringent scientific or medical evidence to back up these actions. These actions are often a result of the rapid dissemination of data and research that were in the preprint stage and had not undergone full peer review [10,11]. We must acknowledge that the influence from online search trends, online social media platform discussions, and dissemination of information is not always reliable and can also be harmful [10].

While social media and online health information sources have almost always universally been appreciated for their potential, they have often not been successfully implemented as key components of public health strategy [12]. Only a small minority of medical and public health researchers would have actively engaged in this space in a professional capacity, as social media has been seen as a means of disseminating information rather than obtaining it [12]. The COVID-19 pandemic has reshaped the use of online platforms from online tools, search engines, and social media sites as potential tools of public health strategy, but also for disease surveillance and monitoring and, indeed, for more immediate transmission of acute changes in disease management [7]. A balance must be struck between the lethargic delay in translational medicine and the impulsive dissemination of as yet–unproven research and information dissemination, in particular during a global health crisis.

### Limitations

This study is limited by the single local center analyzed in Ireland. The search terms and trends utilized were from one social media platform and can only provide a tentative inference of impact on practice. The Google Trends data were only collected on a worldwide sample, as the number of local and regional searches were too small for appropriate analysis. We also note that values from Google Trends are not absolute values of search results, but rather normalized values. However, the use of Google Trends in this study was used to represent a general trend of online discussion that occurred online during the early months of the COVID-19 pandemic, whereby online platforms, such as Google, Twitter, and Facebook, provided platforms for swift data dissemination for health practitioners [13].

### Conclusions

In this paper, we described a phenomenon of local change in clinical practice following worldwide online conversation and digital search trends that influenced individual clinicians before the formation of formal clinical guidelines, with tentative natural improvement and significant improvement following a quality-improvement intervention.

## Abbreviations

CSV: comma-separated values
VTE: venous thromboembolism
